# Supraorbital Rim Fracture Involving Frontal Sinus by Sports Injury

**DOI:** 10.7759/cureus.12003

**Published:** 2020-12-09

**Authors:** Shaul Hameed Kolarkodi, Yasir Alyahya, Muhammad Qasim Javed, Fareed Ahmed Bava, Nishana Mariyam M

**Affiliations:** 1 Maxillofacial Surgery and Diagnostic Science, College of Dentistry, Qassim University, Buraydah, SAU; 2 Conservative Dentistry and Endodontics, College of Dentistry, Qassim University, Buraydah, SAU; 3 Maxillofacial Surgery, Kings Dental Center, Doha, QAT; 4 Oral Pathology and Microbiology, Kings Dental Center, Doha, QAT

**Keywords:** supraorbital rim fracture, sports injury, computed tomography, facial trauma

## Abstract

Supraorbital rim fracture is a rare sports-related injury encountered by craniofacial specialty and great challenge to the surgeons because of their anatomical location and relation with vital structures in close relation. Currently, in the literature, no classification system or treatment protocol exists for the supraorbital rim fracture. Supraorbital rim fracture forms a small proportion of sports-related injuries. Here we present a case with supraorbital rim fracture by sports injury diagnosed by computed tomography and treated surgically by open reduction methods using mini plates.

## Introduction

Fractures of the supraorbital region are rare and are frequently associated with high-energy cranio-maxillofacial trauma. Orbital rim fractures are challenging to manage because of their close proximity to the eye, ocular muscles, underlying frontal sinus, and ultimately the brain. Therefore, during the process of assessment and treatment planning, it is mandatory to evaluate the extent of involvement of the various anatomical structure that lies in close relation to the affected area that includes nerves, eye muscles and cranial base in the anterior cranial fossa (cerebrospinal fluid leakage). Moreover, due consideration should be given to the possible morbidity risk associated with different types of operative approaches while selecting the management approach [1].

Most common etiological factors associated with trauma to the supraorbital region include blow out injuries, personal assault, and road traffic accidents. Overall, one-third of the injuries to human bodies are the result of sports injuries. Facial fractures can involve the supraorbital rims and the anterior table of the frontal sinus in approximately 1 to 9% of cases, and many supraorbital rim fractures are associated with other forms of craniomaxillofacial injury [2]. However, isolated supraorbital rim fracture resulting from a sports injury is a rare phenomenon [1-3]. Here we report a case of 19-year-old male who received a hit from a co-player’s elbow while playing cricket and presented with step deformity in the supraorbital region.

## Case presentation

**Figure 1:**
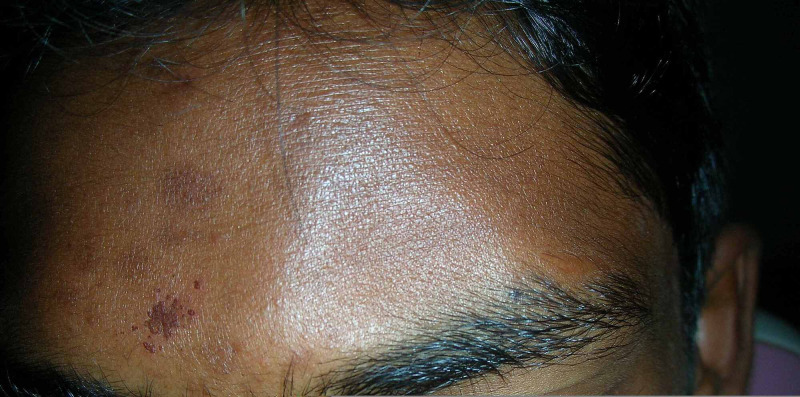
Preoperative clinical picture showing depression above the left eyebrow.

A 19-year-old male patient reported to the department with the chief complaint of pain and discomfort above the left eyebrow area after sustaining a head impact from a co-player’s elbow while playing cricket. The pain started two days after the impact. The medical history of the patient was unremarkable, and he was up to date with his tetanus immunization. The patient did not show any sign of head injury or neurological deficit on examination. The extra-oral examination did not show any asymmetry or swelling other than the depression that was noted on the left supraorbital region. Local examination of the left supraorbital margin revealed bony depression measuring 2 x 1.5 cm that was extending 1 cm medial to the midline, laterally till 2 cm from external eye canthus and superiorly 2 cm from the supraorbital margin (Figure 1).

No subconjunctival hemorrhages/ecchymosis was noted and no abnormality was appreciated on the nose/maxilla. On palpation, mild tenderness was noted at the middle of the left supraorbital margin and there was a step deformity at the middle 1/3rd. However, there were no associated changes in the vision (diplopia) and ocular movements were normal on eye examination. Based on the patient’s history and clinical examination, a provisional diagnosis of left supraorbital margin fracture was made.

**Figure 2:**
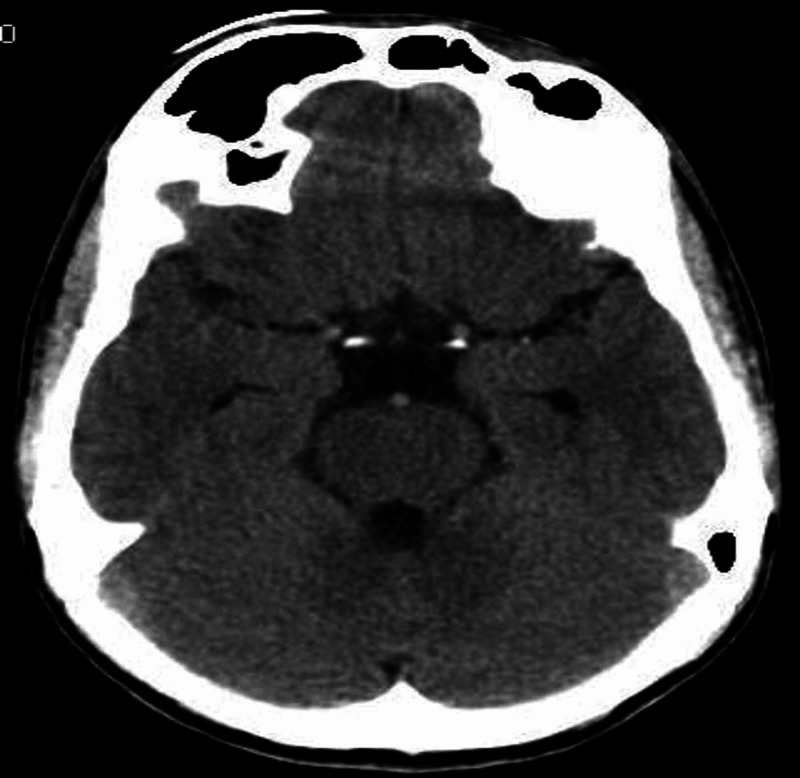
CBCT axial slice showing depressed fracture of left supraorbital bone involving frontal sinus.

**Figure 3:**
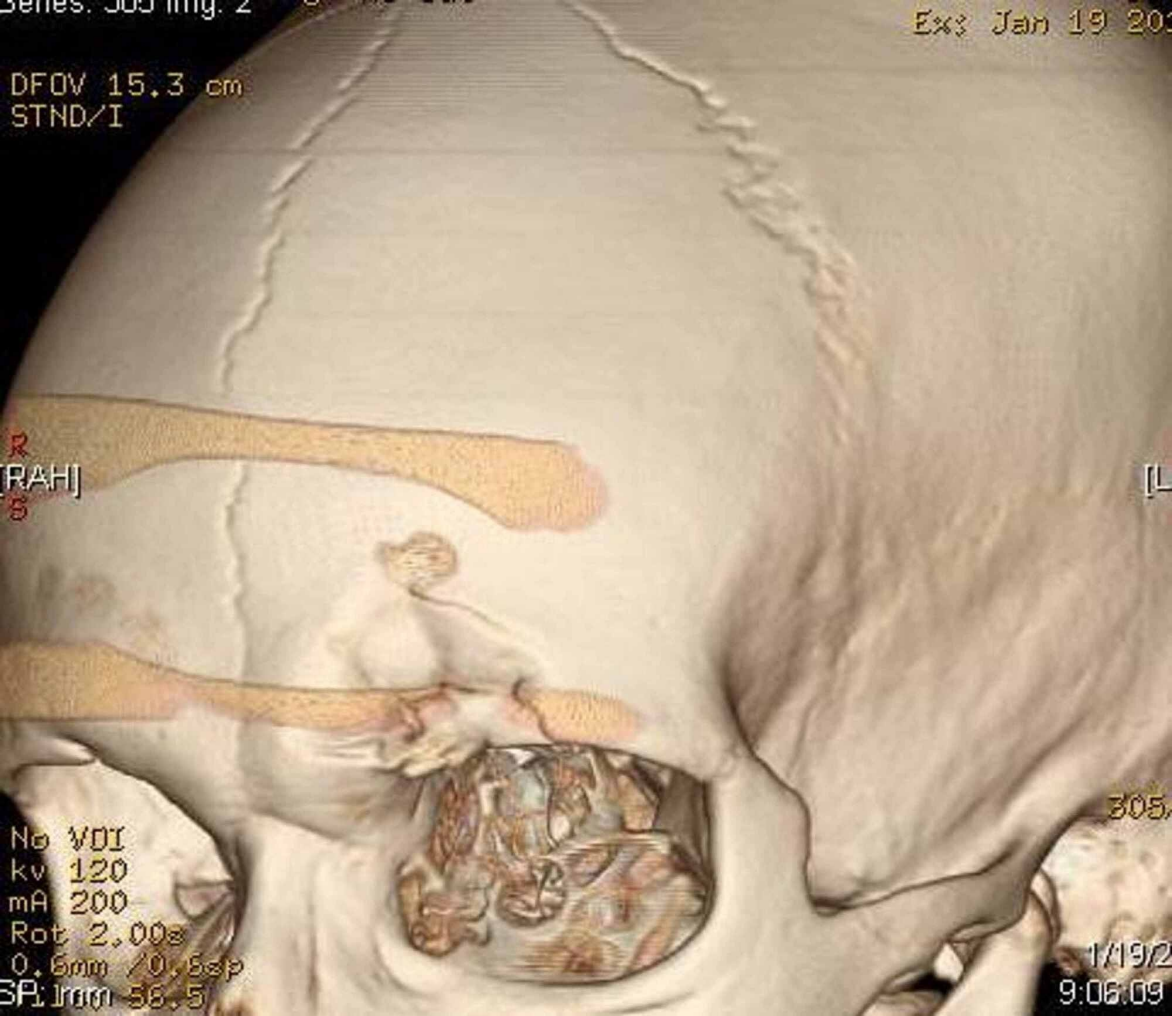
3D reconstructed image showing depressed fracture of the left supraorbital bone.

Paranasal sinus projection with open mouth revealed discontinuity in the left supraorbital margin at the medial 1/3rd and junction of middle and lateral 1/3rd. Also, the haziness was noted in the left frontal sinus suggestive of fluid collection. Computed tomography (CT) scan confirmed the involvement of the frontal sinus (Figures 2, 3).

**Figure 4:**
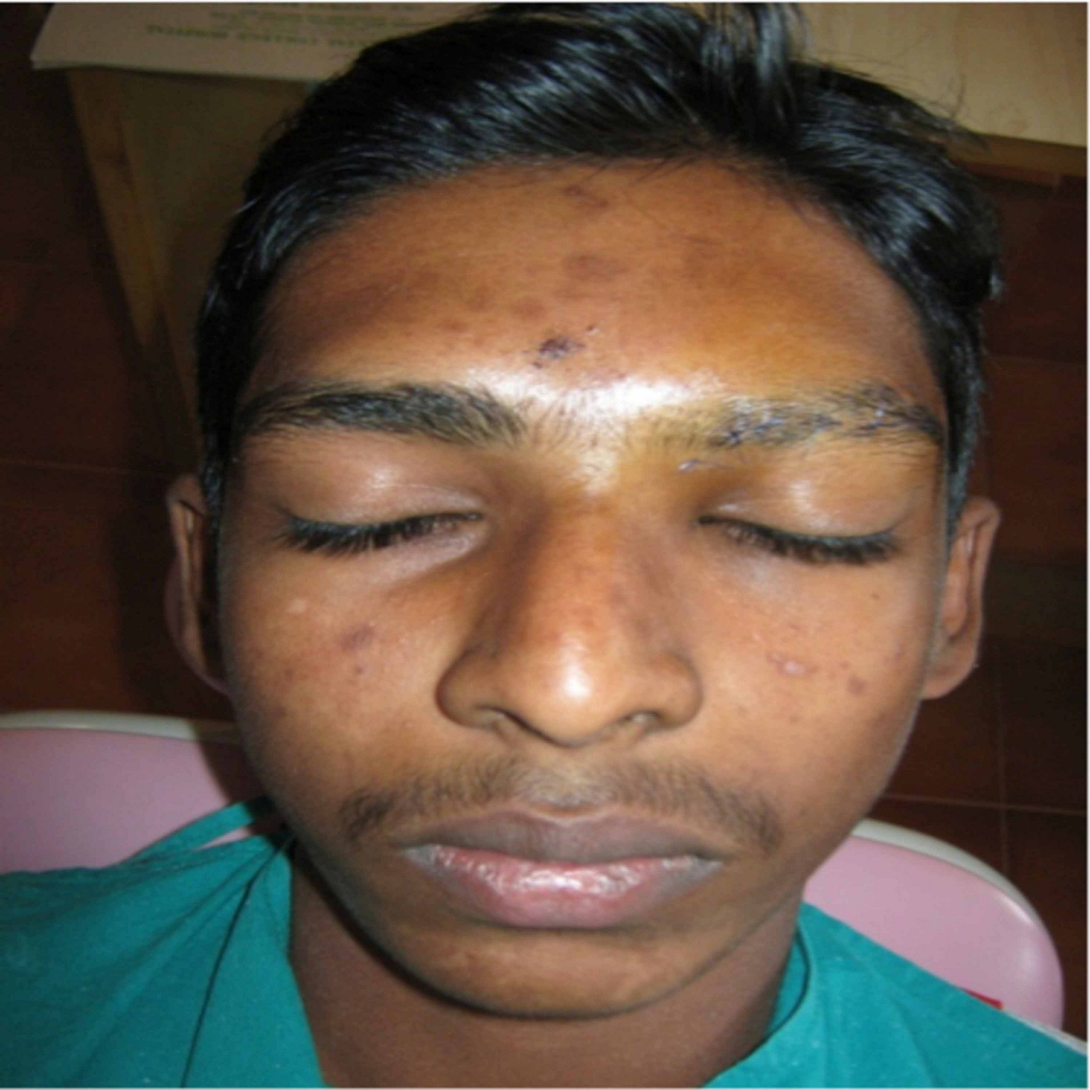
Postoperative picture restoration of the depression with normal appearance.

The patient was informed about the diagnosis with an explanation of possible treatment options. Subsequently, after obtaining consent, the patient was treated surgically by open reduction, and mini plates were fixed with screws. Consequently, the facial aesthetic was restored to normal without any postoperative complication (Figure 4).

## Discussion

Sports injuries account for 4-18% of the facial trauma and facial bone fractures occur in 11-40% of cases [4,5]. The incidence varies according to the type of sports, gender, and age [1-3]. Fractures of the maxillofacial region resulting from sports are more frequent in males and between the ages of 20 and 30 years. This may be attributed to the high level of physical activity in the aforementioned age group, their passion for sports, and participation in dangerous sports while ignoring the consequent risk [3].

It is of foremost importance for the healthcare professionals that are supporting athletes to familiarize themselves with the anatomy of the facial region. Moreover, they should be well aware of the common type of facial injuries, their assessment, and initial management. The most common type of sports-related facial trauma is soft tissue injuries and dentoalveolar fractures and minor facial bone fractures of the nose, zygoma, mandible, and orbital fractures [6-8]. The orbital fractures and zygomatic fractures can threaten the vision. The majority of reported sports-related facial injuries are due to direct hit with a ball (34.1% of cases) or by a collision between players (24.5% of cases) during team sports [4,5,7,8]. The facial bone fractures in team sports usually result from a collision with another player, elbow-head impact, or head-head impact [6,8]. Similarly, the 19-year-old male in the current case sustained a supraorbital rim fracture involving the frontal sinus after being struck in the face by an opponent’s elbow while playing cricket.

The diagnosis of facial fractures is accomplished by the clinical examination and the use of imaging modality. More often, facial deformity from trauma is initially concealed by overlying edema and soft tissue injuries. Therefore, it is important to take a proper history of gauging the severity and mechanism of injury [8,9]. When an injury occurs near the eye, a thorough eye examination should be performed to determine any defects in the vision. During the examination the area in and around the orbit should be palpated. Fractures of the orbital rim can occur at any point on the rim; however, fractures of the inferior rim are most common. Isolated supraorbital rim fractures are rare, and involve the anterior table of the frontal sinus in 1-9% of cases as was evident in the present case [8-10].

Supraorbital rim fractures usually present with cosmetic deformity consisting of depression or flattening of the supraorbital ridge that can be visualized like in the current case. Later the patient may present with soft-tissue edema, lacerations, and periorbital ecchymosis. If the supraorbital and supratrochlear nerves are involved then paresthesia can occur [10]. Present case on palpation revealed a step deformity on the left supraorbital margin, an orbital rim fracture was suspected and for confirming diagnosis the patient was referred for radiographic evaluation. Imaging studies are mandatory to identify/characterize the type/extent of fractures. CT imaging remains the gold standard for detecting and evaluating orbital fractures. Moreover, it helps in the detection of concomitant facial fractures [8,10]. Likewise, a CT scan was done in the present case that revealed the depressed fracture on the left frontal bone in the left supraorbital margin involving the frontal sinus.

The treatment of supraorbital rim fractures should be directed by the functional and esthetic needs of the patient. Open reduction is the treatment of choice in the current case and in most of the displaced supraorbital fracture cases. When there is the displacement of the fractured segments then, surgical exploration, reduction, and stabilization are indicated [8,10]. Supraorbital rim fractures frequently involve the frontal sinus as in our present case. If the anterior table of the frontal sinus and the supraorbital rim is displaced, then operative treatment is required. In our presented case the fracture was treated by open reduction and plating was done to correct the step deformity.

Lastly, the use of protective equipment is the key to prevent facial injuries in sports. It was reported that orbital fracture can almost always be prevented with the use of protective eyewear during sports. The risk of injury to the eye is highly related to the type of sport [1,6]. Hence it is important to educate and create awareness among youth on the use of protective sports gear.

## Conclusions

Orbital rim fractures constitute a small, but significant, number of sports-related facial injuries. This case report highlighted the impact of elbow hit while playing cricket resulting in a supraorbital fracture. There is a need to educate all youths regarding the use of personal protective equipment and adherence to the rules of sports to reduce the incidence of maxillofacial injuries from sports. Analysis of fracture patterns and concomitant injuries is imperative to develop effective management strategies and prevention techniques.
